# Thermal electron-tunneling devices as coolers and amplifiers

**DOI:** 10.1038/srep21425

**Published:** 2016-02-19

**Authors:** Shanhe Su, Yanchao Zhang, Jincan Chen, Tien-Mo Shih

**Affiliations:** 1 Department of Physics, Xiamen University, Xiamen 361005, People’s Republic of China; 2 Beijing Computational Science Research Center, Beijing 100084, People’s Republic of China; 3 Institute for Complex Adaptive Matter, University of California, Davis, CA 95616, USA

## Abstract

Nanoscale thermal systems that are associated with a pair of electron reservoirs have been previously studied. In particular, devices that adjust electron tunnels relatively to reservoirs’ chemical potentials enjoy the novelty and the potential. Since only two reservoirs and one tunnel exist, however, designers need external aids to complete a cycle, rendering their models non-spontaneous. Here we design thermal conversion devices that are operated among three electron reservoirs connected by energy-filtering tunnels and also referred to as thermal electron-tunneling devices. They are driven by one of electron reservoirs rather than the external power input, and are equivalent to those coupling systems consisting of forward and reverse Carnot cycles with energy selective electron functions. These previously-unreported electronic devices can be used as coolers and thermal amplifiers and may be called as thermal transistors. The electron and energy fluxes of devices are capable of being manipulated in the same or oppsite directions at our disposal. The proposed model can open a new field in the application of nano-devices.

Numerous nanoscale studies that are related to harnessing thermal energy focus on pioneering concepts, fundamental principles, and unexplored mechanisms. They are exemplified by photosynthesis^1^,^2^, quantum heat engines^3^,^4^, spin-Seebeck power devices^5^, thermal rectifiers^6^,^7^,^8^, and Brownian motors^9^. Here, we consider a practically-functional device that consists of three electron reservoirs maintained at temperatures, *T*_*h*_, *T*_*c*_, and *T*_*m*_ as well as at chemical potentials, *μ*_*h*_, *μ*_*c*_, and *μ*_*m*_ [Fig. 1(a)], where distributions of electrons filled within these reservoirs obey Fermi-Dirac (FD) statistics, *f*(*ε*, *μ*, *T*)^10^,^11^. These three reservoirs, connected by energy filtering tunnels serving heating, pumping, and feedback functions, establish a continuous cycle [Fig. 1(b,c)]. Such a three-terminal device has functions similar to thermal transistors. The first model of the thermal transistor to control heat flow was proposed by Li *et al.* using Frenkel-Kontorova FK lattices^12^,^13^. Prior to the analysis, let us first define the energy level at the intersection of two given FD distributions as E^*^, yielding 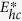, 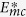, and 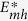
14,15, which denote reversible electron-transport energy levels between each pair of reservoirs. By adjusting three energy levels *E*_*hc*_, *E*_*mc*_, and *E*_*mh*_ relatively to these three starred levels, we are able to determine the directions of electron fluxes and achieve multiple purposes, namely, cooling and thermal amplification.

## Results

**Figure 1:**
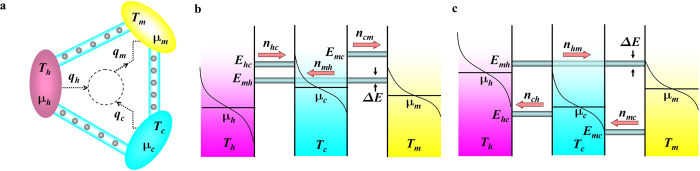
System schematics. (**a**) Thermal electron-tunneling devices that can serve dual purposes of cooling and amplification spontaneously with *T*_*h*_ > *T*_*m*_ > *T*_*c*_; The thermal energy, *q*_*h*_, exits from the hot reservoir, and is used as a power source, which allows the thermal energy, *q*_*c*_, to be released from the cold reservoir, and lets the thermal energy, *q*_*m*_, be pumped to the median reservoir. (**b**) For *μ*_*c*_ > *μ*_*h*_, electron and thermal fluxes flow in the same direction with *E*_*mh*_ being the lowest tunnel energy level. (**c**) For *μ*_*c*_ < *μ*_*h*_, electron and thermal fluxes flow in the opposite direction with *E*_*mh*_ being the highest tunnel energy. Red arrows in (**b**,**c**) indicate directions of electron fluxes.

The thermal devices are designed to remove *q*_*c*_ from the cold reservoir such that the cold reservoir is maintained at the cold temperature, *T*_*c*_, and deliver thermal energy, *q*_*m*_, to the median reservoir. To achieve these purposes, we use *q*_*h*_ as the power source in substitution of the electrical power. Note that *q*_*h*_ and *q*_*c*_ are net positive quantities exiting hot and cold reservoirs, whereas *q*_*m*_ is a net positive quantity entering the median reservoir. The center circle in Fig. 1(a) represents the equivalent results of the thermal energy transportation. In Fig. 1(b), the electron flux, *n*_*ij*_, traveling through the tunnel between two given reservoirs, can be computed by Landauer equation^16^,^17^ as


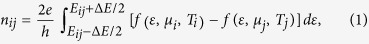


where 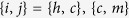, or 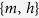. Alternatively, we can write 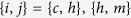, and 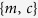
[Fig. 1(c)]. The pre-factor 2 accounts for the degeneracy of electrons; *e* the elementary charge; and *h* the Planck constant. Because the continuity of the electron flux requires that 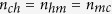, three electron fluxes and thermal energy depend on each other. For example, the change of *μ*_*h*_ and *T*_*h*_ of the hot reservoir will affect the electron flux and thermal energy between the cold and median reservoir. Each electron leaving or entering a reservoir will carry away or inject the thermal energy equaling the difference between its kinetic energy and chemical potential^18^,^19^,^20^. The electron tunneling can now be realized in a semiconductor nanowire with double-barrier resonant-tunneling structure^19^. The energy levels of the all tunnels can be tuned by adjusting the barrier and well widths of the nanowire heterostructure. The thermal flux, *q*_*h*_, associated with electron fluxes *n*_*hc*_ and *n*_*mh*_ can be obtained from Eq. (1) by inserting *ε* − *μ*_*h*_ in the integrand^21^,^22^,^23^ and deleting *e* as


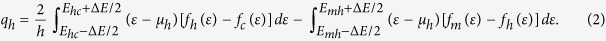


Likewise, we can calculate *q*_*c*_ and *q*_*m*_ using equations similar to Eq. (2). Temperatures considered here lie in the cryogenic range, so that we can neglect the lattice-related thermal conduction^24^,^25^,^26^ and focus on electron kinetic energies^27^,^28^.

**Figure 2:**
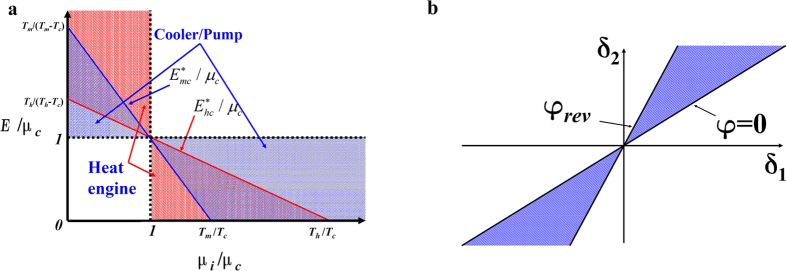
Regime diagrams. (**a**) The operable regime defined by the abscissa *μ*_*i*_/*μ*_*c*_ (*i* = *h* or *m*) and the ordinate *E*/*μ*_*c*_. (**b**) Topography of the operable regime for the proposed device.

Figure 2(a) represents a regime diagram defined by the abscissa *μ*_*i*_/*μ*_*c*_ (*i* = *h* or *m*) and the ordinate *E*/*μ*_*c*_, which can be used to outline the principle of controlling electron-flux directions. Worth noting are four lines, namely, vertical dotted, horizontal dotted, red, and blue lines that represent, respectively, *μ*_*i*_/*μ*_*c*_ = 1, *E*/*μ*_*c*_ = 1, 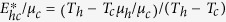, and 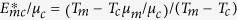. Between hot and cold reservoirs, above the red line and left to the vertical dotted line lies the regime suitable for electron and thermal fluxes moving in the same direction from hot to cold reservoirs; below the red line and right to the vertical dotted line lies the regime suitable for counter flows (hot → cold for energy fluxes and cold → hot for electron fluxes). Between the cold reservoir and the median reservoir, below the blue line and above the horizontal dotted line lies the regime suitable for electron and thermal fluxes traveling from cold to median reservoirs; below the horizontal dotted line and above the blue line lies the regime suitable for counter flows (cold → median for energy fluxes and median → cold for electron fluxes). The vertical dotted line divides the diagram into two regimes: same-direction flows to the left, and counter flows to the right. According to Fig. 2 and analyses above, the systems shown in Fig. 1 are equivalent to the coupling systems composed of energy selective electron Carnot heat engines and coolers.

Next, we describe how to determine directions of thermal fluxes. The first case [Fig. 1(b)] is characterized by *μ*_*c*_ > *μ*_*h*_. The tunnel at the energy level, *E*_*hc*_, connects hot and cold reservoirs. If 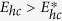, the FD distribution in the hot reservoir is higher than the counterpart in the cold reservoir, implying that the electron flow will spontaneously travel from the hot reservoir to the cold reservoir. Since 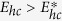 and 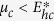 (Supplementary S-1), we can obtain *E*_*hc*_ − *μ*_*c*_ > 0, and *E*_*hc*_ − *μ*_*h*_ > 0. These two inequalities imply that a positive thermal flux leaves the hot reservoir and a positive thermal flux enters the cold reservoir. Next, because *T*_*c*_ < *T*_*m*_, the energy level, *E*_*mc*_ must be lower than 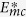. Under this condition, the FD distribution at *E*_*mc*_ in the median reservoir is lower than the counterpart in the cold reservoir, implying that the electron flow will spontaneously move from the cold reservoir to the median reservoir, and that the continuity of the electron flow is satisfied.

Regarding signs of energy fluxes, only when *E*_*mc*_ − *μ*_*c*_ > 0 and *E*_*mc*_ − *μ*_*m*_ > 0, a positive thermal energy leaves the cold reservoir, and a positive thermal flux enters the median reservoir. At this juncture, the only remaining task is the comparison of magnitudes of *μ*_*m*_ and *μ*_*c*_. According to analyses above, we should have 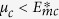. Finally, we obtain *μ*_*c*_ > *μ*_*m*_ (Supplementary S-1), under which the cooler can work. The *E*_*mh*_ level should be designed such that the continuity of electron fluxes is guaranteed. Therefore, we are able to utilize this condition to determine *E*_*mh*_ numerically. Once having made this determination, we are able to obtain *q*_*c*_ exiting the cold reservoir and the thermal flux *q*_*h*_ leaving the hot reservoir. Subsequently, we are able to determine the cooling performance. This mechanism can work for thermal amplification processes as well if our interest lies in deliver thermal energy to the median reservoir.

The second case [Fig. 1(c)] is characterized by *μ*_*c*_ < *μ*_*h*_ and we can design a cycle whose characteristics are similar to those in the first case, but the electron and thermal fluxes flow in the opposite direction (Supplementary S-2). Figure 1(c) can also be designed to work as a cooler or an amplifier. We also observe that *μ*_*c*_ < *μ*_*m*_ (Supplementary S-2).

## Discussion

### Thermal electron-tunneling device as a cooler

When the thermal device works as a cooler, we can define the cooling modulus as *φ* = *q*_*c*_/*q*_*h*_. As Δ*E* → 0, we can obtain *q*_*c*/*h*_ in a simplified form based on Eq. (2) as


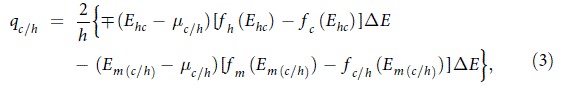


where symbols 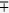 correspond to cases of subscripts *c* and *h*, respectively. Because continuity equations of electron fluxes satisfy 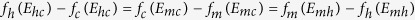, we obtain the cooling modulus as


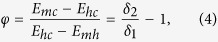


where *δ*_1_ = *E*_*hc*_ − *E*_*mh*_ and *δ*_2_ = *E*_*mc*_ − *E*_*mh*_. When *E*_*hc*_, *E*_*mc*_, and *E*_*mh*_ equal 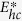, 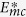, and 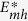, respectively, we obtain^29^,^30^


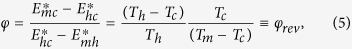


implying that electron transports via three tunnels are reversible, and the cooler yields a reversible performance, *φ*_*rev*_.

From Eq. (4), we can conclude that *δ*_1_ and *δ*_2_ are two crucial independent parameters to determine the performance of the device. As indicated in Fig. 2(b), *φ* is required to be larger than zero by suitably selecting values of *δ*_1_ and *δ*_2_, lying within the shaded area confined by *φ* = 0 and *φ* = *φ*_*rev*_. For ideal tunnels whose electron occupations of states are infinitesimally close to an equilibrium state and whose widths become infinitesimally small, reversible electron transports can be achieved (Supplementary S-3).

**Figure 3:**
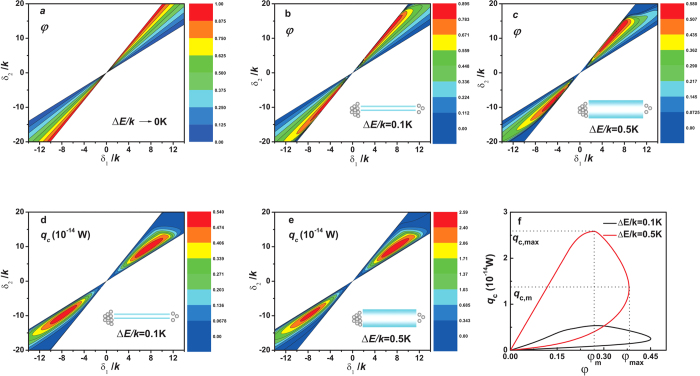
Cooling performance of the proposed device. (**a**–**c**) Cooling modulus, *φ*, as a function of *δ*_1_ and *δ*_2_, parametrized in the tunnel width, Δ*E*/*k*. (**a**) As Δ*E*/*k* approaches zero, the device attains the reversible performance. (**b**,**c**) As Δ*E*/*k* increases, the irreversibility related to the electron transport increases, causing *φ* to decrease. (**d**,**e**) The cooling rate, *q*_*c*_, as a function of *δ*_1_ and *δ*_2_. We are able to identify *q*_*c*,*max*_ in the operable regime. (**f**) *q*_*c*_ versus *φ* after optimization of *q*_*c*_ with respect to *δ*_1_. There exists a negative slope arc segment on which *q*_*c*_ decreases as *φ* increases. This trend appears more pronounced for Δ*E*/*k* = 0.5 *K*.

For non-ideal cases, tunnel energy levels deviate from 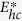, 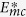, and 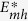 and their widths become finite. For purposes of illustrating performance characteristics, let us choose *T*_*h*_ = 3 *K*, *T*_*m*_ = 1.5 *K*, *T*_*c*_ = 1 *K*, and *μ*_*c*_/*k* = 10, as shown in Fig. 3. The parameter *μ*_*h*_/*k* will be optimally designed in the following discussion. The other parameter *μ*_*m*_/*k* will be computed through electron-flux continuity equations.

Figure 3 reveals the performance of the thermal device as a cooler. The results in Fig. 3 are simultaneously determined by *μ*_*c*_, *μ*_*h*_, *μ*_*m*_, *E*_*hc*_, *E*_*mh*_, *E*_*mc*_, and Δ*E*. The parameter, *μ*_*h*_, has been optimized for maximum *q*_*c*_, while *μ*_*m*_ has been designed to satisfy continuity equations of electron fluxes (Method). Figure 3(a–c) show contour plots of the cooling modulus, *φ*, versus *δ*_1_ and *δ*_2_, parameterized in Δ*E*/*k* approaching 0 *K*, or equaling 0.1 *K* and 0.5 *K*. Values of *φ* are seen to be approximately symmetrical to the point at *δ*_1_/*k* = 0 *K* and *δ*_2_/*k* = 0 *K*. For *μ*_*c*_ > *μ*_*h*_ [Fig. 1(b)], results show that *δ*_1_ > 0 and *δ*_2_ > 0, indicating that *E*_*mh*_ should be adjusted to the lowest. For *μ*_*c*_ < *μ*_*h*_ [Fig. 1(c)], we find that *δ*_1_ < 0 and *δ*_2_ < 0, implying that *E*_*mh*_ should become the highest. For this configuration, the electron flux and the energy flux cross each other.

In Fig. 3(a), Δ*E*/*k* → 0, *φ* appears to be a monotonic function of *δ*_1_ and *δ*_2_. When the maximum value of the cooling modulus, *φ*_max_, approaches unity, which is computed from Eq. (5) for ideal cases, the model will exhibit its reversible performances. When the tunnel width becomes finite [Fig. 3(b,c)], contours show maxima. For example, when Δ*E*/*k* = 0.5 *K*, *φ*_*max*_ approaches 0.580, which is lower than the reversible value. The area enclosed by the innermost contour in Fig. 3(b) is smaller than that in Fig. 3(c), suggesting that it is easier to select *δ*_1_ and *δ*_2_ to achieve *φ*_max_ in the case shown in Fig. 3(c). As Δ*E*/*k* widens, tunnels lose their abilities to select electrons, leading to electron-transport irreversibilities, thus lowering *φ* values.

The value of *q*_*c*_ increases as Δ*E*/*k* increases, but the irreversibility also increases [Fig. 3(d,e)]. However, we can find that maximum *q*_*c*_ exists with respect to *δ*_1_ and *δ*_2_. Alternatively, we optimize *q*_*c*_ with respect to *δ*_1_ and obtain *q*_*c*_ as a function of *φ* [Fig. 3(f)]. The *q*_*c*_ versus *φ* curve is a closed loop passing through the origin. On the curve, there exists a *φ*_*max*_ whose corresponding cooling rate is *q*_*c*,*m*_, and a maximum cooling rate *q*_*c*,*max*_ whose corresponding coefficient is *φ*_*m*_. Thus, the ranges of the cooling modulus and the cooling rate must be constrained by *φ*_*m*_ ≤ *φ* ≤ *φ*_*max*_ and *q*_*c*,*m*_ ≤ *q*_*c*_ ≤ *q*_*c*,*max*_. Clearly, *φ*_*max*_ and *q*_*c*,*max*_ determine upper bounds of the cooling modulus and the cooling rate, while *φ*_*m*_ and *q*_*c*,*m*_ give lower bounds of the optimized values of both. When the cooler is operated in the optimally working region with negative slope arc segments, both the cooling rate, *q*_*c*_, and the rate of the entropy production of three electron reservoirs, 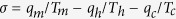, are of monotonically decreasing functions of the cooling modulus, *φ*. For example, it can be obtained from the negative slope arc segment with Δ*E*/*k* = 0.5 K in Fig. 3(f) that when *φ* = 0.268, *q*_*c*_ = 2.582 × 10^−14^ *W* and *σ* = 2.357 × 10^−14^ *W*/*K*; when *φ* = 0.333, *q*_*c*_ = 2.204 × 10^−14^ *W* and *σ* = 1.470 × 10^−14^ *W*/*K*. Thus, one should simultaneously consider both the cooling modulus and the cooling rate in the practical design of devices.

### Thermal electron-tunneling device as an amplifier

When the thermal device works as an amplifier, the amplification ratio 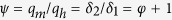. Following similar arguments described for coolers, as Δ*E*/*k* approaches zero and *E*_*hc*_, *E*_*mc*_, and *E*_*mh*_ equal 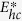, 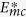, and 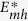, respectively, we can derive the amplifier ratio as 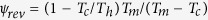
31, yielding the reversible performance. For general cases, one can discuss the performance of an amplifier by using the similar method analyzed for a cooler. This shows that such a device can behave as a cooler or an amplifier, depending on our interest in extracting thermal energy from the cold reservoir, or pumping thermal energy into the median reservoir.

The proposed model is one of thermal spontaneous conversion devices that have been rarely searched. By optimizing the energy levels of all tunnels in the FD sense, we are capable of manipulating flux-directions at our disposal, constructing either coolers or thermal amplifiers in the absence of electrical power inputs, and concurrently reducing flux irreversibilities to achieve high thermal performances. The pioneering investigation on the proposed model can open a new avenue for building practical thermal electron-tunneling devices and have potentially significant applications where thermal manipulation at micro/nano levels is required.

## Methods

All the integrals are numerically performed by using the Gaussian Quadrature. For given values of *μ*_*c*_ and Δ*E*, there are five unknown parameters, namely, *μ*_*h*_, *μ*_*m*_, *E*_*hc*_, *E*_*mh*_, and *E*_*mc*_. The numerical method to evaluate the device performance is summarized as follows: (1) By transforming the abscissa (horizontal, *δ*_1_) and the ordinate (vertical, *δ*_2_) into *E*_*hc*_ = *δ*_1_ + *E*_*mh*_ and *E*_*mc*_ = *δ*_2_ + *E*_*mh*_, we are left with three unknowns: *μ*_*h*_, *μ*_*m*_, and *E*_*mh*_. (2) When *μ*_*h*_ is further given, the continuity equations 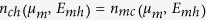 and 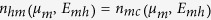 are numerically computed self-consistently to obtain *μ*_*m*_ and *E*_*mh*_. For solving these two nonlinear integral equations, we loop the two unknown variables. One loop is nested in the other loop. The inner loop is stopped if 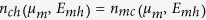 is valid, and then we check the other equation. If 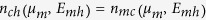 is not valid, we go back to the outer loop. This procedure is repeated until two continuity equations are valid. (3) Following the above calculation, we optimize *μ*_*h*_ such that the thermal energy *q*_*c*_ reaches maxima. (4) With the help of all parameters precisely determined, the cooling modulus *φ* can be obtained. In semiconductors, the position of *μ* relative to the band structure is usually controlled by doping with donor and acceptor impurities^10^. The biased chemical potentials can also be generated by the external electric powers, but the consumption of electricity must be considered to evaluate the device performance.

## Additional Information

**How to cite this article**: Su, S. *et al.* Thermal electron-tunneling devices as coolers and amplifiers. *Sci. Rep.* **6**, 21425; doi: 10.1038/srep21425 (2016).

## Supplementary Material

Supplementary Information
