# Supplementary material for: Thermal electron-tunneling devices as coolers and amplifiers
Source: Sci Rep. 2016 Feb 19;6:21425. doi: 10.1038/srep21425 (PMC4759785; doi:10.1038/srep21425)
Supplement: Supplementary Information [file srep21425-s1.pdf]

Supplementary information for thermal electron-tunneling devices as coolers and amplifiers

Shanhe Su[1,2], Yanchao Zhang[1], Jincan Chen[1], Tien-Mo Shih[3]

[1]Fujian Key Laboratory of Semiconductor Materials and Applications and Department of Physics, Xiamen University, Xiamen 361005, People's Republic of China

[2]Beijing Computational Science Research Center, Beijing 100084, Peoples Republic of China

[3]Institute for Complex Adaptive Matter, University of California, Davis, CA 95616, USA

In this supplementary, relationships among reversible tunneling energy levels and chemical potentials of reservoirs are examined. The principle of the second case (Fig. 1c) with $\mu_c < \mu_h$ will also be analysed in detail. The reversible performance of the thermal electron-tunneling device that operates as a cooler will be computed. Finally, we will show that the system satisfies both the first and second laws of thermodynamics:

1. The reversible tunneling energy level

It has been proved that, if Fermi-Dirac probability distributions of two reservoirs intersect at the energy level of a tunnel, electron transports are guaranteed to be reversible. Based on this proof, we can design heat engines, pumps, or coolers by placing tunneling levels below or above E_{hc}^{*}

$$E^*_{hc} = \frac{T_h\mu_c - T_c\mu_h}{T_h - T_c} = \mu_c + \frac{T_c\left(\mu_c - \mu_h\right)}{T_h - T_c} = \mu_h + \frac{T_h\left(\mu_c - \mu_h\right)}{T_h - T_c}. \qquad \text{(S1)}$$

According to Eq. (S1), it is found that $\mu_c < E^*_{hc}$ and $\mu_h < E^*_{hc}$ under conditions of $T_h > T_c$ and $\mu_c > \mu_h$. On the other hand, $\mu_c > E^*_{hc}$ and $\mu_h > E^*_{hc}$ hold if $T_h > T_c$ and $\mu_c < \mu_h$.

2. The second case (Fig. 1c) characterized by $\mu_c < \mu_h$

If $E_{hc} < E^*_{hc}$, Fermi-Dirac distribution $f_h(E_{hc})$ in the hot reservoir is lower than the counterpart in the cold reservoir $f_c(E_{hc})$, implying that the electron flow will spontaneously travel from cold to hot reservoirs. From Eq.(S1), we obtain $\mu_c > E^*_{hc}$ and $\mu_h > E^*_{hc}$, such that, from analyses above, $E_{hc} - \mu_c < 0$, and $E_{hc} - \mu_h < 0$. Hence, a negative thermal energy leaves the cold reservoir, and a negative thermal flux enters the hot reservoir. We observe that these two fluxes cross each other. Consequently, the former will gain energy; the latter will lose energy. The electron flux can be withdrawn to benefit the next subsystem, leading to the fact that this pair behaves as a heat engine.

If $E_{mc} > E^*_{mc}$, Fermi-Dirac distribution at $f_c(E_{mc})$ in the cold reservoir is lower than the counterpart in the median reservoir $f_m(E_{mc})$, implying that the electron flow will move from the latter to the former to guarantee the continuity of electron flows. This phenomenon requires $E_{mc} - \mu_c < 0$ and $E_{mc} - \mu_m < 0$. As a result, a negative thermal energy leaves the median reservoir, and a negative thermal flux enters the cold reservoir, such that the median reservoir will gain energy, and the cold reservoir will lose energy. From analyses above, we have $\mu_c > E^*_{mc}$. Combining

with Eq. (S1), we will obtain $\mu_c < \mu_m$. Therefore, the pair of the cold reservoir and the median reservoir behaves as a heat pump. This hybrid model of the heat engine and the heat pump can be regarded as an amplifier or cooler.

3. Reversible performance of the amplifier

When the thermal electron-tunneling device works as an amplifier, the amplification ratio, ψ, is defined as

$$\psi = q_m / q_h . \tag{S2}$$

As $\Delta E \to 0$, we can obtain $q_{m/h}$ in a simplified form based on Eq.(2) as

$$\begin{aligned} q_{m/h} = \frac{2}{h}\{ & \pm(E_{(m/h)c} - \mu_{m/h})(f_c(E_{(m/h)c}) - f_{m/h}(E_{(m/h)c}))\Delta E \\ & -(E_{mh} - \mu_{m/h})(f_m(E_{mh}) - f_h(E_{mh}))\Delta E\} \end{aligned} , \tag{S3}$$

where symbols $\pm$ correspond to cases of subscripts m and h, respectively. Because the continuity equations of electron fluxes satisfy

$$f_h(E_{hc}) - f_c(E_{hc}) = f_c(E_{mc}) - f_m(E_{mc}) = f_m(E_{mh}) - f_h(E_{mh}) , \tag{S4}$$

it can be found from Eqs. (S2)-(S4) that the amplification ratio is given by

$$\psi = \frac{q_m}{q_h} = \frac{E_{mc} - E_{mh}}{E_{hc} - E_{mh}} . \tag{S5}$$

When E_{hc}, E_{mc}, and E_{mh} equal E_{hc}^*, E_{mc}^*, and E_{mh}^*, respectively, we obtain

$$\psi = \frac{E_{mc}^* - E_{mh}^*}{E_{hc}^* - E_{mh}^*} = \frac{T_h - T_c}{T_h} \frac{T_m}{T_m - T_c} \equiv \psi_{rev} , \tag{S6}$$

implying that electron transports via three tunnels are reversible, and the thermal electron-tunneling device yields a reversible performance, ψ_{rev}.
